# PsyHELP pocket guide: Development of an educational tool to promote professional mental health help-seeking among the health workforce

**DOI:** 10.1371/journal.pone.0309224

**Published:** 2024-10-04

**Authors:** Muhammad Syafiq Kunyahamu, Aziah Daud, Tengku Alina Tengku Ismail, Mohd Faiz Md Tahir

**Affiliations:** 1 Department of Community Medicine, School of Medical Sciences, Universiti Sains Malaysia, Kota Bharu, Kelantan, Malaysia; 2 Department of Psychiatry, Kulliyyah of Medicine, International Islamic University Malaysia, Kuantan, Pahang, Malaysia; Stanford University, UNITED STATES OF AMERICA

## Abstract

**Introduction:**

Ensuring the mental well-being of the health workforce is important in maintaining a robust healthcare system. This paper aims to describe the development of PsyHELP pocket guide and its potential to encourage the health workforce to recognise and seek help from mental health professionals for their mental health concerns.

**Method:**

Developed with the Health Belief Model (HBM) as its theoretical framework, this PsyHELP pocket guide integrates theoretical and practical strategies, employing a user-centric design that combines text, visuals, and interactive elements, such as QR codes linked to animation videos, to enhance engagement and accessibility. The content development involved a thorough literature review and was structured to align with the HBM, addressing various constructs that influence help-seeking.

**Result:**

The PsyHELP pocket guide series, conceptualised as multiple pocket guides, begins with foundational information about mental health and progresses to offer actionable strategies tailored for the health workforce. It addresses vital mental health concepts, combating stigma, recognising the need for professional help, and providing steps towards mental well-being, ensuring a comprehensive approach to mental health awareness and action among the health workforce.

**Conclusion:**

The PsyHELP pocket guide stands out as a promising resource, aiming to enhance mental health awareness and encourage help-seeking behaviours among the health workforce, fostering a supportive and mentally healthy work environment.

## Introduction

Maintaining good mental health is crucial for everyone as it profoundly affects an individual’s quality of life, productivity, and overall well-being. The importance of mental health has gained global attention in recent years, leading to various initiatives aimed at enhancing mental well-being and addressing mental health problems [1]. Notably, the health workforce is among the group of workers who are vulnerable to mental health problems, potentially influenced by various factors, such as the nature of their work, societal expectations, and the rigorous emotional and physical demands they face [2, 3].

It is imperative for individuals, especially those in the health workforce, to seek professional help when confronted with mental health challenges. However, navigating the complex and multifaceted mental health landscape demands a delicate balance between professional demands and societal expectations. Several factors, including a strong preference for self-reliance, a low perceived need for help, stigma, and a lack of awareness, often hinder the health workforce from seeking help, leading to unaddressed mental health concerns that are perpetuated over time [4–7].

Health workforce often finds their mental health overlooked or deprioritised, a scenario further complicated by the inherent pressures of their roles and the challenges of navigating a healthcare system [2]. This not only impacts individual well-being but also is crucial for sustaining the overall efficacy and resilience of healthcare systems [3]. Furthermore, numerous recent studies show a notable prevalence of mental health problems among the Malaysian health workforce, underscoring the urgent need for targeted strategies [8–10]. Local data indicates a substantial treatment gap, with only 14.2% of healthcare workers seeking professional help for mental health issues despite high levels of stress and mental health challenges [11]. These findings are consistent with international studies showing low help-seeking rates among healthcare professionals, such as those by Fridner *et al*. [12], Grover *et al*. [13], and She *et al*. [14]. Locally, a study in Putrajaya, Malaysia, found that only one-third of Malaysian civil servants had a moderate intention to seek counseling services for mental health concerns, while the remaining two-thirds displayed a low level of intention Chong *et al*. [15]. As such, the development of resources that not only educate them about mental health but also inspire them to seek help when necessary is essential.

Considering the need for resources tailored to the local context, the PsyHELP pocket guide has been conceptualised and developed. This guide aims to empower the health workforce with knowledge and strategies to prioritise their mental well-being, navigate challenges, and seek timely and appropriate help from mental health professionals. This paper describe the development of the PsyHELP pocket guide and its potential to encourage the health workforce to seek help from mental health professionals.

## Methodology

### Theoretical framework

The PsyHELP pocket guide was developed using the Health Belief Model (HBM) as its foundational theoretical framework, a well-established theoretical framework in health behaviour research [16–18]. The HBM suggests that an individual’s health-related behaviour is influenced by their perceptions and beliefs, encompassing several main constructs: perceived susceptibility and severity, perceived benefits, perceived barriers, cues to action, and self-efficacy.

In the context of this PsyHELP, the constructs of perceived susceptibility and severity highlight the mental health risks faced by the health workforce and their potential consequences. The perceived benefits construct emphasises the positive outcomes of seeking mental health support, focusing on an individual’s belief in the efficacy of the advised action to mitigate risks. Central to the perceived barriers construct is the challenge of stigma, a significant deterrent preventing many health workforces from seeking assistance. The cues-to-action construct encompasses actionable strategies and resources to promote mental health help-seeking, while self-efficacy bolsters health workforces’ confidence in their ability to seek and benefit from professional help.

The decision to use the HBM as the guiding framework was driven by its widespread application in various health contexts, including health behaviours related to mental health [16, 19]. The HBM’s emphasis on individual perceptions and beliefs makes it particularly suited for addressing mental health help-seeking, where personal beliefs play a pivotal role in determining whether or not an individual seeks help. Moreover, the HBM’s focus on perceived barriers aligns well with the challenges faced by the health workforce, especially in contexts where stigma around mental health is prevalent.

Furthermore, the decision to utilise the HBM as the guiding framework was reinforced by findings from our prior unpublished study conducted among 470 health workforces in the East Coast Region of Peninsular Malaysia that identified two main factors that are associated with mental health help-seeking: perceived need for help and perceived stigma barrier [20]. These findings not only aligned with the HBM’s constructs of perceived severity and benefits as well as perceived barriers but also highlighted the unique challenges faced by health workforce in the Malaysian context. This alignment between empirical findings and theoretical constructs provided a robust foundation for the guide, ensuring that it is both theoretically grounded and empirically informed.

### The PsyHELP structure design

The PsyHELP pocket guide design was crafted with a pronounced emphasis on its end-user, the health workforce, which encompasses a broad range of individuals including those directly involved in clinical services, such as doctors, nurses, pharmacists, and psychologists, as well as those in supportive roles, such as administrative staff, accountants, and ambulance drivers. Acknowledging their demanding schedules and the challenges they encounter, the guide was created in a way that would be both informative and actionable. Decisions regarding language, format, and additional features such as QR codes were made to ensure the guide’s user-friendliness, conciseness, easily accessible, and resonance with the cultural and linguistic nuances of the Malaysian health workforce.

Layman’s terms were used to ensure effective communication with readers, translating complex concepts into easily digestible and applicable information. The guide also integrated a blend of text, visuals, and interactive elements, using eye-catching design and colours to further enhance engagement. QR codes linked to animation videos were incorporated to leverage modern technology and cater to diverse learning preferences, providing a dynamic and illustrative overview of information. Presenting information in a concise yet impactful way is crucial to ensure that it is accessible and memorable for the readers [21].

Overall, the pocket guide design process harmoniously blended theoretical underpinnings, empirical insights, and practical considerations, resulting in a guide that is both academically robust and pragmatically useful for health workforces.

### Content development process

The development of the PsyHELP pocket guide began with a thorough review of literature on mental health help-seeking, barriers to seeking help, and effective interventions. Studies were sourced from prominent databases such as PubMed, Google Scholar, and Scopus using search terms related to mental health, help-seeking, health workforce, and barriers. Additionally, authoritative resources and publications from renowned organisations like the World Health Organization (WHO), the American Psychological Association (APA), and the Malaysia Ministry of Health were reviewed to ensure that the guide’s content aligned with global and local recognised standards and practices.

The guide’s content was meticulously structured with the HBM as the guiding framework and insights from our previous research on the two factors associated with mental health help-seeking intention: the perceived need for help and the perceived stigma barrier. Each section of the guide was crafted to align with a specific construct of the HBM, ensuring a comprehensive approach to addressing the myriad factors influencing help-seeking. The decision regarding the number and title of the guide series was made to provide a comprehensive and holistic approach to mental health. The guide series begins by addressing basic mental health concepts and gradually progress to offer actionable strategies specifically tailored for the health workforce. This approach aimed to empower health workers to be proactive in their mental well-being journey.

The emphasis during the development process was particularly placed on overcoming stigma and recognising the need for help, given their identification as primary associated factors in our prior study. The interactive nature of the guide, coupled with real-life case studies, was chosen to foster engagement and reflection, ensuring that readers are not just passive consumers of information but active participants in their mental health journey. The development process was iterative, involving continuous refinement to ensure clarity, relevance, and alignment with the intended objectives of promoting mental health awareness and help-seeking among health workforces.

## Result

The PsyHELP pocket guide is a meticulously structured guide tailored for the health workforce, addressing their mental health challenges. It encompasses several series, each focusing on a specific aspect of mental health, from foundational knowledge to actionable strategies.

The PsyHELP pocket guide series comprises four separate books, each focusing on a specific aspect of mental health. Each book contains approximately 20–25 pages, ensuring concise yet comprehensive coverage of topics. The animation videos were developed by the research team using the Renderforest website, chosen for its user-friendly interface, flexibility, and array of customizable options. This online tool allowed for the creation of professional-quality animated videos without requiring advanced technical skills, making it an ideal choice.

### The PsyHELP format and design

The guide’s physical presentation was decided to be in an A6 size book format, chosen for its portability and convenience for the workers to carry or store it in their workspaces. Each book in the series was organised with a clear title and series number, enabling quick navigation for readers. The guide was primarily written in Malay, an official language of Malaysia, ensuring cultural resonance and accessibility. Each main series chapter also included animation videos that were created to provide a dynamic and illustrative overview of information. These videos are integral to enhancing understanding and engagement, offering an alternative learning modality for users. Access to these animation videos is facilitated through strategically placed QR codes within the guide.

### The PsyHELP content

The PsyHELP series, presented as a collection of pocket guides, commenced with Book 1, entitled “Basic Information on Mental Health: What You Need to Know.” This initial guide serves as an essential introduction for the health workforce, offering vital foundational insights into mental health. The guide’s foreword provides a glimpse of what readers could expect from the entire series, from understanding basic mental health concepts to addressing stigma and recognising the need for professional help, thus bridging the gap between awareness and action. Book 1 further delved into the fundamental concepts of mental health and its overarching significance in daily life, exploring the intricate relationship between mental and physical well-being and emphasising how mental health can influence various facets of life, from workplace productivity to interpersonal relationships. This section aligned with the HBM’s perceived susceptibility and severity constructs, educating readers about the potential risks and consequences of neglecting mental well-being.

Following the series is Book 2, “Combating Stigma”, designed to equip readers with an in-depth understanding of the origins, manifestations, and impacts of stigma. This edition confronted common misconceptions and stigma associated with mental health, empowering readers to counteract these biased views. The content aligned with the HBM’s perceived barriers construct, offering actionable insights to overcome these barriers. To further enrich the content, this chapter incorporated an inspirational true story, providing a real-world perspective on the challenges and triumphs related to mental health. A QR code was also embedded within the pages, granting readers access to an animated video that offered a dynamic and illustrative overview of the chapter’s key concepts.

Book 3, titled “Professional Help: Why is it Needed?”, emphasised the importance of recognising the need for professional help. It aimed to enhance readers’ ability to recognise when professional mental health support is necessary and to comprehend the significance of seeking such help. The book outlined common indicators signalling the necessity for professional involvement, aligning with the HBM’s construct of perceived benefit. Book 3 also delved into the rationale behind seeking help, shedding light on its pivotal role in mental health care. To enrich the reader’s experience and facilitate a deeper connection with the material, the book featured an inspirational true story. Additionally, a QR code was incorporated, providing access to an animated video that further elucidates the chapter’s concepts, thereby enhancing engagement and comprehension.

Book 4, entitled “From Understanding to Action”, serves as the series’ call to action. This book provides a pragmatic roadmap, guiding readers from recognising symptoms to seeking professional assistance and underscores the importance of cues to action and self-efficacy, key constructs of the HBM. The concept of “MUDAH” was introduced as a structured approach towards achieving mental well-being. Additionally, this book offered valuable advice and tips to help readers prepare for their initial consultations with mental health professionals. A comprehensive resource section was also included, listing available mental health services and helplines, ensuring that readers had readily available necessary resources. The inclusion of QR codes linked to animated videos further enriches the content, offering diverse learning avenues and making the information more engaging and accessible. The series concluded by summarising its key messages, emphasising the importance of mental health awareness, challenging stigma, and the importance of timely intervention.

To provide a clear overview of the content and its connection to the Health Belief Model (HBM) elements, Table 1 detailing the content, subthemes, and corresponding HBM elements for each book is included below.

**Table 1:** Content overview of the PsyHELP pocket guide’s series and connection to health belief model.

Book Title	Content/Subthemes	Connection to HBM Elements	Animation Videos
Book 1: Basic Information on Mental Health	Definitions, Importance, Mental and Physical Well-being	Perceived Susceptibility, Severity [29]	Introductory Video on Mental Health
Book 2: Combating Stigma	Misconceptions, Impacts of Stigma, Overcoming Stigma	Perceived Barriers [29]	Video on Stigma and Its Effects
Book 3: Professional Help: Why is it Needed?	Indicators for Seeking Help, Benefits of Professional Support	Perceived Benefits [29]	Video on When and Why to Seek Help
Book 4: From Understanding to Action	Steps to Seeking Help, "MUDAH" Concept	Cues to Action, Self-Efficacy [29]	Video on Steps to Seeking Help

## Discussion

The development and conceptualisation of the PsyHELP pocket guide come at the right moment in the evolving field of mental health, aiming to bridge the gap between awareness and proactive help-seeking behaviours, specifically tailored to meet the needs of the health workforce. This initiative aligns with recommendations for effectively addressing mental health problems, which include raising mental health awareness, dismantling barriers to accessing care, reducing stigma related to seeking and receiving mental health care and encouraging the utilisation of professional services [2, 3, 22]. The strategic development of resources like the PsyHELP pocket guide can play a role in encouraging individuals to seek mental health support while also ensuring that the information and guidance provided are delivered in a way that is not only cost-effective and affordable but also inclusive and accessible to any health workforce.

The guide offers numerous potential impacts and benefits to enhance the mental health support landscape for the health workforce. Firmly grounded in the HBM, this guide serves as a valuable resource, aiding them in navigating through the complexities of mental well-being with clarity and purpose. The guide not only addresses vital mental health concerns but also emphasises understanding and combating stigma, fostering a more inclusive environment where the health workforce can seek help without fear of judgement or discrimination. Furthermore, by highlighting the signs of mental health issues and the importance of timely intervention, the guide encourages the health workforce to seek help at an early stage, potentially preventing more severe mental health challenges.

Highlighting the importance of seeking help is pivotal, especially given the dedicated nature of the health workforce, which often prioritises others’ well-being above their own. While this dedication is often seen as admirable, it can be detrimental if it prevents workers from seeking necessary help for their own well-being. Furthermore, some workers might fear that engaging physically in mental health programs could lead to discrimination or stigmatisation [5, 23, 24]. In this context, a written educational self-guided tool like PsyHELP provides a unique resource. Unlike group workshops or programmes, the PsyHELP pocket guide ensures easy dissemination of information to the target audience and enables workers to privately explore information about mental well-being in their own time and space, thereby mitigates fears of exposure or judgment and provides a stigma-free environment.

This approach is more practical than attendance-required programs, which might be challenging for busy workers. It is also difficult to ensure that everyone in the healthcare sector is involved in such programme. Furthermore, this subtle approach not only respects the privacy and autonomy of the health workforce but also subtly challenges the stigma around mental health by positioning professional help and information-seeking as normal and positive actions. As highlighted by Bernier [25], educational materials offer advantages such as message consistency, reusability, portability and reinforcement of information thereby maximising people’s knowledge and adherence to treatment. However, the appropriate readability level of these materials is crucial for maximum effectiveness and meet the needs of the health workforce.

The guide’s potential lies not only in its informative content but also in its potential to change the way mental health is perceived and addressed among the health workforce. The introduction of easy-to-recall concepts like “MUDAH” (which translates to “easy” in Malay) ensures that the guide is not only theoretically robust but also pragmatically actionable. Serving as a mnemonic, “MUDAH” guides the health workforce through a series of steps to prioritise and address their mental well-being, simplifying the process and providing a ready reference point to enhance the guide’s practicality and usability. This aligns with the suggestion that initiatives should primarily focus on promoting the positive aspects of mental health in a manner that is easily comprehensible and accessible to individuals [2].

A pivotal decision in the design process was the use of the Malay language, ensuring that the content resonated with the cultural and linguistic nuances of the Malaysian health workforce. This aimed to enhance the guide’s accessibility and relatability, ensuring that its primary audience can easily understand and internalise the content. Additionally, incorporating QR codes that link to animated videos provides a multimodal learning experience, catering to diverse learning preferences and leveraging modern technology to enhance the guide’s interactivity and contemporaneity. The use of simple language, complemented by relevant illustrations, ensures that the content is easily digestible, catering to both clinical and non-clinical health workforce, regardless of their prior knowledge of mental health.

Within the extensive array of available mental health resources, the PsyHELP distinguishes itself by specifically focusing on the health workforce suitable for the local context in Malaysia. While most mental health intervention strategies or educational tools, such as Minda Sihat Module and KOSPEN Plus initiative in Malaysia, adopt a broad approach aimed at catering to a wide audience, their universal nature may potentially dilute the effectiveness of such interventions for specific groups like the health workforce. Universal interventions have not consistently shown significant changes in help-seeking behaviour, thus emphasising the need for targeted interventions that are specifically tailored to the targeted audience [26]. This is different from the PsyHELP pocket guide, which specifically focuses on the health workforce. This specificity fosters a deeper connection, making the content more relatable and instilling a sense of belonging among the health workforce, making them feel seen, understood, and valued.

In the broader context of mental health support, a multitude of services have emerged to assist individuals in managing psychological issues, utilising mechanisms such as peer support groups and dedicated phone support lines. Malaysia’s “Heal 1555” hotline is an example, a service that offers online psychological support to anyone, including the health workforce [27]. However, accessing this service often requires a level of intention and awareness about the need to seek help, which can be a barrier for some individuals. Yu *et al*. [28] pointed out that people will not utilise mental health services if they have no intention to seek mental health assistance. The PsyHELP pocket guide aims to bridge this gap by empowering individuals and enhancing their autonomy over health-related decisions. This approach has the potential to motivate even those initially hesitant to become more engaged in their mental health journey.

### Recommendations and future directions

The development and potential of the PsyHELP pocket guide signifies a promising initial step towards enhancing the mental health of the health workforce. While the guide is meticulously crafted, it has yet to undergo user feedback and content validation by a panel of experts. Future studies should incorporate these steps to ensure the guide’s practical applicability and effectiveness. This step is needed to ensure that the content is theoretically robust and empirically substantiated in its practical applicability and efficacy in real-world settings.

Future endeavours should prioritise a thorough validation process, ensuring that the guide not only aligns with the needs and preferences of its target audience but also resonates with the expertise of mental health professionals and specialists in health workforce well-being. This validation should encompass not only the content but also probe into the guide’s usability and pragmatic effectiveness in real-world scenarios. Gaining insights into the users’ perspectives, identifying challenges, and pinpointing areas for enhancement will be instrumental in refining the guide, ensuring it remains pertinent and user-centric.

While the current format of the guide is designed to be practical and accessible, future iterations could explore additional digital resources and technological integrations. For example, incorporating links to online platforms, helplines, or even augmented reality features could enhance user engagement and experience. The exploration into digital elements, such as dedicated apps or online communities, is suggested as a future direction to further support the health workforce in their mental well-being journey, providing them with a holistic and multifaceted support system.

## Conclusion

In conclusion, the PsyHELP pocket guide stands as a beacon of hope, promising a brighter, more informed, and supportive environment for the health workforce to navigate the mental health challenges of their profession. While the guide represents a significant step forward in addressing the mental well-being of the health workforce, it is an initial step. Continuous refinement and validation will be essential to ensure its sustained relevance and effectiveness.
